# Narrating anxiety as “ordinary”: digital self-healing diaries and the normalization of anxiety on Chinese social media

**DOI:** 10.3389/fpsyg.2026.1795151

**Published:** 2026-05-22

**Authors:** Yuying He, Xudong Zhou

**Affiliations:** 1 Anhui University, Hefei, China; 2 South China Normal University, Guangzhou, China

**Keywords:** anxiety, China, illness narrative, normalization, social media

## Abstract

Anxiety disorders are highly visible on Chinese social media, where users document symptoms, treatment, and everyday struggles through “self-healing diaries.” Yet research has paid limited attention to how these patient-authored narratives construct the meaning of anxiety in digital settings. Drawing on thematic narrative analysis, this study examines 80 self-healing diaries posted on Xiaohongshu to explore how anxiety is narratively normalized in online self-disclosure. The analysis identifies four recurrent narrative modes: acceptance, coexistence, compromise, and opportunity. Acceptance narratives reframe anxiety as a manageable and acceptable condition; coexistence narratives embed anxiety within the routines of everyday life; compromise narratives de-exceptionalize anxiety by locating suffering within broader human and social conditions; and opportunity narratives refigure anxiety as a meaningful challenge and a possible pathway to growth. Rather than treating anxiety solely as a medical problem or an exceptional crisis, these narrative modes reposition it within an ordinary framework of self-understanding and daily life. By showing how digital self-healing diaries normalize anxiety through narrative meaning-making, this study contributes to scholarship on illness narratives, mental-health communication, and platform-mediated self-disclosure in contemporary China.

## Introduction

Anxiety disorders are among the most prevalent mental disorders in China, with a lifetime rate of 7.6% and a 12-month rate of 5.0% (Huang et al., 2019). Their social and emotional visibility extends beyond clinical settings and is now evident across Chinese social media platforms, where anxiety is named, discussed, and interpreted through both everyday expressions and psychiatric terminology. Trends such as “madness literature,” anti-involution discourse, relaxation, and “lying flat” reflect the broad circulation of anxiety-related feelings in contemporary China (Zhang and Li, 2023). Meanwhile, clinical terms—such as social phobia, obsessive-compulsive disorder, generalized anxiety disorder, and somatization—have also gained visibility online. Rather than functioning only as channels for information exchange, digital platforms have become important spaces in which mental distress is publicly narrated, interpreted, and negotiated, even as they may also intensify psychological pressure (Schønning et al., 2020).

Psychiatric and clinical psychological research has generated important knowledge about the diagnosis and treatment of anxiety disorders (Sareen et al., 2005; Sik, 2021). However, clinical knowledge alone cannot fully explain how individuals themselves make sense of anxiety in everyday communicative practice. The rise of self-care and patient-centered approaches in mental health has drawn greater attention to patients’ own accounts of illness experience (Lucock et al., 2011). Within health communication and illness narrative research, such accounts are important not because they can be equated with therapy, but because they show how symptoms, suffering, treatment, and selfhood are interpreted outside clinical encounters and circulated in social life (Frank, 1995; Gray, 2009; Li and Xu, 2023; Xu and Li, 2023). Examining patient-authored narratives therefore helps illuminate how meanings of illness are formed, stabilized, and shared in communicative settings that are public, peer-oriented, and digitally mediated.

Against this background, Chinese social media “self-healing diaries” offer a particularly revealing form of platform-mediated illness narration. In these posts, users document symptoms, consultations, medication experiences, emotional fluctuations, and reflections on everyday life. Narrative is central here not merely as a rhetorical form, but as a means of organizing experience and assigning meaning to illness (Fisher, 1985; Sharf and Vanderford, 2003). Although digital mental-health narratives have received growing scholarly attention, fewer studies have examined how Chinese anxiety patients use self-authored diary-like posts to narratively normalize anxiety—that is, to reposition it from an exceptional crisis to a more ordinary, manageable, and meaningful part of daily life. This article therefore examines how anxiety is normalized through narrative meaning-making in Xiaohongshu self-healing diaries, and how different recurrent narrative modes make such normalization possible.

## Literature review

### Illness narratives, meaning-making, and the normalization of anxiety

Narrative has long been understood in health communication as more than a stylistic feature of discourse. It is a practical and interpretive mode through which individuals organize experience, assign causality, and render disruption meaningful in ways that can be communicated to others (Fisher, 1984, 1985; Gray, 2009). In illness contexts, narratives do not merely recount symptoms or events; they also reveal how sufferers position illness in relation to the self, to everyday life, and to socially available ways of understanding distress (Frank, 1995; Sharf and Vanderford, 2003). This perspective is especially useful for the study of anxiety, as it directs attention away from anxiety as a clinical label alone and toward anxiety as an experience that is narrated, interpreted, and socially situated.

A central function of illness narratives is meaning-making. When illness unsettles familiar assumptions about the body, the future, and the self, storytelling may help reorganize this disruption into coherent sequences and intelligible interpretations (Dervin, 1998; Aronowitz, 1998; Bury, 2001). Previous research has shown that illness narratives can facilitate sense-making, assert control, transform social roles and identities, warrant decisions, and build community (Arntson and Droge, 1987; Sharf and Vanderford, 2003). These functions are not merely individual in scope or effect. In co-narrated or collectively circulated stories, sufferers may validate one another’s pain and generate shared experiential knowledge (Bülow, 2004). Communication-based narrative theories likewise emphasize that storytelling links communication to health and well-being by enabling individuals to interpret stress, anxiety, worry, and depression through socially meaningful frames (Koenig Kellas, 2018). Narratives, then, are not simply retrospective representations of illness; they participate in the process through which illness becomes understandable, sayable, and livable.

Recent scholarship has shown that patient-authored mental-health narratives in digital environments offer particularly rich access to this interpretive work. In Chinese online depression communities, for example, sufferers draw on recurring discursive patterns to make sense of pain, evaluate the self, and construct explanations of illness through narrative interaction (Li and Xu, 2023; Xu and Li, 2023). Related studies further indicate that such communities are not merely repositories of distress, but spaces in which shared meanings, identities, and communities of practice are actively formed (Li and Xu, 2025a). Identity reconstruction may itself become a major outcome of such narrative participation, as sufferers reinterpret depression in ways that render continued life and selfhood more coherent (Li and Xu, 2025b). Taken together, these studies suggest that digital patient narratives constitute patterned and socially meaningful forms of data for narrative analysis rather than isolated or purely expressive statements.

Within this broader line of inquiry, normalization may be understood as a narrative process through which distress is repositioned within the horizon of ordinary life rather than treated solely as rupture or exception. Anxiety may be narrated not only as disruptive or overwhelming, but also as something that can be named, interpreted, endured, and incorporated into everyday existence. Research on health disruption likewise suggests that communication can contribute to the reconstruction of a “new normal” when prior understandings of health and selfhood are unsettled (Genuis and Bronstein, 2017). Other studies show that narratives may challenge dominant illness representations and support the reconstruction of selfhood and identity (Cooper, 2021; Gunning and Koenig Kellas, 2024). From this perspective, narrative can be approached not only as a mode of meaning-making but also as a mode of normalization, through which suffering becomes more intelligible, more shareable, and, in some cases, more manageable within everyday life.

### Digital mental-health narratives on social media

While illness narratives have long occupied an important place in health communication, digital platforms have altered the conditions under which such narratives are produced, circulated, and interpreted. Social media has expanded the public visibility of mental distress and created spaces in which users narrate symptoms, compare explanations, seek recognition, and negotiate the meaning of suffering in interaction with peers and broader audiences. Users’ posts, reflections, and self-descriptions have therefore become valuable materials for mental-health research not only because they can be computationally analyzed, but also because they provide naturally occurring accounts of distress, coping, and self-understanding (Chancellor and De Choudhury, 2020; Wongkoblap et al., 2017). Recent research has further shown that social media engagement may shape mental-health outcomes through cognitive and evaluative processes such as social comparison (Le Blanc-Brillon et al., 2025). Social media is thus not merely a channel for the dissemination of mental-health information; it is also a communicative environment in which mental-health meanings are publicly produced, circulated, and contested.

A growing body of research has examined online mental-health narratives as forms of peer communication and interpretive support. Depression blogs, for instance, can bridge the isolation of illness and the openness of public space, allowing sufferers to narrate distress while collaboratively seeking coherence (Kotliar, 2016). Vlogging and other forms of online self-disclosure may likewise reduce social isolation, enhance self-efficacy, and foster a sense of community among people living with mental illness (Sangeorzan et al., 2019). In Chinese contexts, online support groups also function as narrative spaces in which patients exchange advice and rework their illness experiences through interaction with others (Pun and Yu, 2023). These studies are particularly relevant because they show that digital patient narratives are neither purely private texts nor simple reflections of clinical discourse; rather, they are socially situated communicative acts through which suffering is interpreted, negotiated, and shared.

At the same time, recent research suggests that mental-health narratives on social media are neither uniform nor invariably oriented toward recovery. Some studies indicate that social media engagement may, under certain conditions, support adaptive coping even when associated with heightened anxiety or psychological distress (Chen et al., 2025; Jin et al., 2025). Personal accounts of anxiety and depression on YouTube, for example, display recurring thematic patterns while revealing the diversity of ways in which sufferers frame distress, recovery, and identity (Huang et al., 2024). Other work complicates affirmative readings even further. Anonymous distress narratives on social media may emerge precisely because certain forms of suffering are too negative to be heard in ordinary interpersonal settings (Yeo, 2021). Such narratives do not simply promote coping or resilience; they may also testify to despair, communicative breakdown, and the difficulty of making mental suffering tellable. This broader perspective is important because it situates positive reframing as one possible narrative logic among others, rather than as the defining feature of digital mental-health expression.

Platform specificity is equally important. Xiaohongshu is not simply another online health forum. As a lifestyle-oriented platform structured around visibility, relatability, and self-presentation, it encourages a form of public self-disclosure that differs from anonymous support forums or explicitly therapeutic spaces. Recent research on depression-related self-presentation posts on Xiaohongshu shows that users do not merely list symptoms; they construct recognizable identities through recurring themes such as treatment, emotional experience, optimism, and resilience (Gu and Gao, 2025). This line of research is especially relevant because it brings together platform logic, self-presentation, and mental-health narration within a Chinese social media environment.

Against this background, one gap remains particularly significant: namely, how Chinese anxiety patients use diary-like “self-healing” posts on a lifestyle-oriented platform to narratively situate anxiety within everyday life. Existing studies have generated valuable insight into depression communities, support groups, anonymous distress narratives, blogs, vlogs, and self-presentation posts. Less attention, however, has been paid to how anxiety is rendered more manageable, ordinary, or meaningful through self-authored diary narratives. It is precisely this process that the present study examines through an analysis of the recurrent narrative modes found in Xiaohongshu self-healing diaries.

## Method

### Data source and sampling

Xiaohongshu is a rapidly growing lifestyle-oriented social media platform in China, where users share short videos and image-text posts known as “notes” (ThePaper, 2024). Because these notes often combine self-disclosure, everyday reflection, and interaction with other users, the platform has become an important space in which mental distress is publicly narrated and discussed. Anxiety-related topics circulate widely on Xiaohongshu, and many users document symptoms, consultations, medication experiences, and daily struggles in posts explicitly framed as “self-healing diaries.” Such diary-like posts are especially relevant to the present study because they foreground patients’ own efforts to interpret anxiety in relation to treatment, selfhood, and everyday life.

Xiaohongshu provides two primary routes for information retrieval: keyword search and topic-based navigation. Using both routes, this study searched for posts with the keyword “anxiety disorder self-healing diary” and filtered the results through the platform’s “most popular” ranking. Candidate texts were selected according to three criteria: first, the author indicated patient status by presenting consultation or treatment records in the account; second, the post contained more than 300 Chinese characters; and third, the post displayed a recognizable narrative sequence broadly consistent with Labov’s (1972) narrative elements, including orientation, complicating action, evaluation, and outcome. Here, Labov’s (1972) narrative elements were used as a practical sampling heuristic rather than as the formal analytic framework of the study. This criterion helped us distinguish diary-like posts with a minimally developed narrative sequence from fragmentary reflections or purely affective expressions. Through four rounds of collection, 80 diary-like posts were retained as the final corpus. Each post was assigned an identifier; for example, C2 refers to the second post collected in the third round.

The individual post served as the sampling unit. Analytically, however, the study focused on the narrative sequences and interpretive segments within posts through which authors explained anxiety, evaluated treatment, and situated suffering in relation to everyday life. Accordingly, the recurrent categories identified in the findings refer to narrative modes that emerge across posts rather than to mutually exclusive post-level genres. Because the corpus was constructed through the keyword “self-healing diary” and the platform’s “most popular” ranking, it represents a visible and publicly shareable subset of anxiety narration on Xiaohongshu, one especially oriented toward coping, reflection, and self-interpretation. It should therefore be understood as a particular narrative formation within the platform environment rather than as an exhaustive representation of all anxiety-related discourse online.

### Analytic approach

This study employed thematic narrative analysis to examine how anxiety is interpreted and given meaning in self-healing diaries. Following Riessman (2008), thematic narrative analysis emphasizes the content of stories rather than their formal structure, asking what is being said, how experiences are linked, and what kinds of meaning are produced through narration. At the same time, it retains narrative sequence as an important analytic principle, since the order in which events, evaluations, and reflections are presented helps illuminate how narrators make suffering intelligible. This approach was particularly suitable here because the study was concerned not simply with isolated expressions or keywords, but with how authors organized anxiety into broader accounts of illness, treatment, and everyday life.

Coding was conducted using MAXQDA 2020. By the 52nd post, no new codes emerged; five additional posts were then analyzed to confirm saturation. Analysis proceeded in four stages. First, initial coding closely followed the diary texts and marked narrative sequences related to illness interpretation, treatment experience, self-evaluation, and everyday coping. Second, focused coding used memos to group recurring codes into more conceptually meaningful categories. For example, repeated references to normal test results, bodily intactness, or limited physical harm were grouped into a broader category concerning the preservation of bodily normalcy. Third, axial coding examined how these categories clustered around broader ways of framing anxiety—for instance, as manageable, livable, socially common, or potentially meaningful. Fourth, thematic synthesis compared provisional categories across the corpus in order to identify recurrent narrative modes while preserving internal coherence and sensitivity to narrative context. In this process, normalization functioned as a sensitizing concept rather than as a fixed code: we examined how posts repositioned anxiety as more acceptable, livable, less singular, or more meaningful within everyday life, and identified the four recurrent modes through comparison of these narrative routes across the corpus. All posts were coded in the original Chinese by the first author. The coding structure, category definitions, representative excerpts, and English translations were then reviewed by the second author, who also checked the translations against the original Chinese posts. Disagreements were resolved through discussion until consensus was reached.

### Ethics

This study complies with relevant ethical standards. Because the analyzed posts were publicly accessible and not subject to access restrictions, they were treated as available for research use (Tripathy, 2013). Ethical review and approval were not required for this study in accordance with local legislation and institutional requirements. To protect privacy, all usernames mentioned in the article are pseudonyms. Given the sensitive and potentially identifiable nature of the narratives analyzed here, we would only share the underlying data for academic purposes.

### Findings

Anxiety disorders are often marked by persistent worry and fear, experiences that may intensify sufferers’ attention to bodily sensations, future uncertainty, and the meaning of illness itself (American Psychiatric Association, 2013). In the self-healing diaries analyzed here, however, anxiety is not narrated in a single, uniform way. Across posts, authors repeatedly draw on a limited set of interpretive patterns through which anxiety is explained, evaluated, and situated in relation to everyday life. The analysis identifies four recurrent narrative modes—acceptance, coexistence, compromise, and opportunity—through which anxiety is variously framed as acceptable, livable, socially common, or potentially meaningful. In this sense, what becomes “ordinary” is not anxiety as such, but the place anxiety is given within everyday self-understanding and daily life. Taken together, these modes show how self-healing diaries narratively reposition anxiety within a more ordinary horizon of everyday experience.

### Acceptance narratives: rendering anxiety acceptable

For many narrators, anxiety initially appears as an unfamiliar and potentially alarming condition. In response, self-healing diaries frequently draw on comparison as an interpretive strategy: anxiety is measured against other bodily illnesses, more severe psychiatric conditions, or prior expectations of what it means to be “really ill.” Through such comparisons, anxiety is not denied, but reclassified as something more understandable and more acceptable to live with. In this narrative mode, normalization operates less by erasing suffering than by reducing its catastrophic force. Anxiety is rendered acceptable insofar as it is framed as physically limited, psychiatrically manageable, and compatible with continued everyday functioning.

#### Affirming bodily normalcy

A first way in which acceptance narratives render anxiety more acceptable is by affirming the body as fundamentally intact. Across many self-healing diaries, narrators contrast anxiety with terminal or chronic physical illnesses and, in doing so, relocate its threat away from bodily damage and toward a more limited sphere of distress. This comparison does not eliminate suffering, but it reduces the catastrophic force attached to anxiety by presenting it as a condition that leaves the body essentially unharmed. In this sense, what is normalized is not suffering itself, but the bodily meaning attached to anxiety: it is narrated less as a threat of physical collapse than as a distressing yet physically limited condition. Within this framing, the confirmation of bodily normalcy becomes a key narrative resource through which anxiety is made more manageable and less alarming.

Health checkups play a central role in this process. Recalling medical examinations and normal test results is a recurring narrative strategy through which authors reassure themselves that no serious bodily illness is present. In such accounts, physical intactness functions as evidence that anxiety, however distressing, does not amount to irreversible physical collapse. For example, C8 described undergoing three cardiac monitoring exams before deciding not to worry anymore: “I used to worry about having all kinds of problems. I even did 24-hour heart monitoring three times. It cost a lot. The results were almost the same each time, so it means there’s nothing serious.” B17 equated physical health with overall well-being more directly: “I’ve already ruled out any organ problems—am I supposed to bury a healthy body?” Within this narrative framework, anxiety is redefined as “just a thought” (A1), distinguishing it from “real illness.”

#### Downscaling cognitive and behavioral disturbance

A second, related way in which acceptance narratives render anxiety more acceptable is by downscaling the severity of its cognitive and behavioral consequences. In this mode, self-healing diaries contrast anxiety with other psychiatric conditions in order to frame it as a form of distress that remains manageable, recoverable, and compatible with reflective self-control. What is being reduced here is not the existence of suffering itself, but the extent to which anxiety is narrated as psychically disorganizing or behaviorally overwhelming. By preserving a sense of rational agency, these accounts reposition anxiety less as a radical break with ordinary mental life than as a condition whose disturbances can still be interpreted, moderated, and worked on.

Many narrators demonstrate basic awareness of psychiatric categories and use such comparisons to differentiate anxiety from forms of mental suffering perceived as more severe or less controllable. For example, B13 recounted a bipolar friend’s repeated breakdowns and self-harm, expressing concern about her own worsening condition and comorbidity. However, she derived comparative comfort: “If I’m afraid my cognition is wrong—if cognition is just a way of thinking—why not think in a better direction?” A13 described anxiety as a “mindset choice,” emphasizing the controllability and non-pathological nature of the condition. In these accounts, anxiety is not denied, but presented as a condition that still allows room for observation, adjustment, and self-direction.

#### Reassuring trust in psychiatric care

A further way in which acceptance narratives render anxiety more acceptable lies in rebuilding trust in psychiatric care. For many narrators, anxiety is initially accompanied by hesitation toward psychiatric medication, concern about dependence, or uncertainty about whether professional treatment is truly necessary. Within self-healing diaries, however, firsthand experiences of medication, clinical consultation, and institutional care are often narrated in ways that make psychiatric treatment appear dependable, effective, and emotionally reassuring. In this mode, normalization operates not by minimizing anxiety itself, but by relocating it within a framework of intelligible and manageable care. Anxiety becomes more acceptable insofar as it is presented as something that can be handled through ordinary medical intervention rather than as an exceptional or uncontrollable crisis.

The immediate effect of medication prompts some patients to reevaluate the practical value of psychiatric care. D1, who had long relied only on psychotherapy and resisted medication, experienced significant improvement after starting pharmacotherapy. He reflected in his diary: “Had I known meds worked this well, I wouldn’t have silently suffered for so long.” In these narratives, psychiatric medications (e.g., Alprazolam) are reframed as “routine medical tools”, equivalent to cold remedies (B17). Such comparisons do not simply praise medication; they work to reduce the symbolic distance between psychiatric treatment and more familiar forms of everyday care.

Although most patients had no experience with hospitalization, a few hospital-related narratives described the orderliness and safety of clinical settings. C7 gave a detailed account of treatment and daily life during admission:

*Three doses of medication a day, afternoon physical therapy (like rTMS), and occasional psychotherapy. Life in the hospital was easy. We’d chat and exercise. The doctor adjusted meds based on my condition, and nurses were attentive. I only had a mild episode on the first night.*

In these accounts, clinical order itself becomes reassuring. Once anxiety is placed within professional routines, it no longer appears as an unbounded threat, but as something that can be monitored, treated, and lived through. Although insurance-related comments did not mention hospitalization, they similarly conveyed reassurance by emphasizing the affordability and accessibility of treatment systems.

### Coexistence narratives: living with anxiety in everyday life

Coexistence narratives normalize anxiety by embedding it within the routines and continuities of everyday life. Rather than presenting anxiety as a disruption that suspends living, self-healing diaries in this mode frame it as something that must be accommodated, managed, and carried alongside ordinary activities. What is emphasized here is not the disappearance of symptoms, but the capacity to continue living with them. Through this narrative logic, anxiety is repositioned less as an exceptional crisis than as a condition that coexists with work, family life, treatment, and ordinary experience. In this sense, normalization takes the form of integration: anxiety is made livable by being woven into the texture of everyday life rather than set apart from it.

#### Embedding anxiety in everyday routines

At the level of everyday practice, coexistence narratives make anxiety livable by absorbing it into the mundane routines of daily life. Although anxiety may disrupt ordinary rhythms and make treatment physically and emotionally taxing, self-healing diaries in this mode do not present illness management as a suspension of life. Instead, they reframe treatment, self-care, and symptom monitoring as tasks that coexist with cooking, cleaning, working, exercising, and caring for others. What is normalized here is not the disappearance of distress, but its incorporation into the repetitive texture of daily existence. Anxiety remains present, yet it is narrated as something that can be carried alongside ordinary responsibilities rather than as a force that wholly overtakes life.

C6 offers a representative account:

*Spring, summer, autumn, winter. Three meals a day. That’s how everyone lives. I just try to do my job well and devote myself to life—even if it’s just cooking a meal for my family, cleaning, organizing the closet, exercising, or taking care of the kids. It’s like my neighbor who goes to the hospital every day to bring food to his father. Everyone is doing some small things every day.*

Here, life is deconstructed into a series of mundane and repetitive units of action. Illness management is no longer narrated as a sudden or exceptional event, but as a routine task on a par with cooking or exercising. In this framing, illness-related activities are woven into the ordinary framework of daily life, so that what comes to the fore is not the disruptive force of anxiety, but the continuity of living in its presence. These narratives do not deny anxiety; rather, they position it as something that can be absorbed into everyday practice and thus made more livable.

#### Revaluing everyday experience

At the level of self-evaluation, coexistence narratives make anxiety livable by shifting value away from ideal outcomes and toward ongoing experience. When anxiety is tied to perfectionism, self-surveillance, and the pressure to recover quickly, it can easily become another measure of personal failure (Mendoza et al., 2024). In this narrative mode, self-healing diaries interrupt that logic by redefining what counts as a meaningful life: rather than judging life in terms of flawless performance, immediate cure, or social proof, narrators place greater value on process, feeling, and participation in experience itself. Anxiety is not thereby celebrated, but it is repositioned within a life that remains worth inhabiting even under conditions of incompleteness and uncertainty.

Diaries record how some patients, after letting go of self-imposed standards and social expectations, reconstruct their criteria for evaluating life. For instance, B2 wrote: “I don’t need to do everything well, nor prove everything to others. If I think I’m good enough, then I am.” When life’s value is reconstructed as being related to personal feeling rather than performance outcomes, patients stop fixating on whether treatment brings immediate results. B19 even bluntly declared: “Let go of the fantasy that anxiety disorder will be cured instantly.”

Letting go of expectations and embracing the diversity of experience allows patients to rediscover the richness of daily life. A travel experience led A2 to realize that interactions with strangers can be a source of joy. She continued seeing her therapist regularly while also joining book clubs, hobby groups, and city walks. She wrote: “I try my best to break through the invisible walls of life and let myself feel this society full of surprises.” In these accounts, the value of life is relocated from achievement to experience. Rather than eliminating anxiety, such narratives make room for it within an expanded sense of living, in which uncertainty, pleasure, treatment, and self-exploration can coexist.

### Compromise narratives: situating anxiety within shared vulnerability

Compromise narratives normalize anxiety by locating it within broader conditions of human limitation, social pressure, and shared vulnerability. Rather than presenting anxiety as a singular or exceptional affliction, self-healing diaries in this mode frame it as one expression of difficulties that are widely distributed across human life. What is emphasized here is not recovery through mastery, but a reduction in the sense that anxiety marks a uniquely personal failure. Through this narrative logic, suffering is de-exceptionalized: anxiety is repositioned as part of a more general condition of living under uncertainty, constraint, and loss of control. In this sense, anxiety becomes less a private anomaly than one manifestation of a broader condition of human and social vulnerability.

#### Locating anxiety beyond personal responsibility

In one strand of compromise narratives, anxiety is reframed less as a matter of personal failure than as a condition shaped by forces beyond the self. When anxiety is narrated as the result of childhood conditions, inherited sensitivity, or other forces outside the self, the illness is no longer framed primarily as evidence of individual weakness or failure. What is reduced here is not suffering itself, but the moral burden attached to it. By shifting the source of anxiety away from culpable selfhood, these narratives loosen the pressure of self-blame and make room for a more resigned, less outcome-driven relation to treatment. In this way, anxiety is repositioned within a broader field of human vulnerability, where its causes are understood as only partly subject to personal control.

In self-healing diaries, recollections of childhood are a frequent strategy for emphasizing uncontrollable causality. For example, A3 believed his anxious disposition stemmed from an unstable family environment: “I think it was the lack of love in my childhood that made me feel extremely unsafe.” Other patients attributed their condition to physiological or genetic factors. B4 suggested her anxiety was related to an inherited sensitivity: “About 15%–20% of people in the world have sensitive temperaments.” Through these narratives, anxiety is framed as an externally conditioned predicament, and being ill is correspondingly removed from a framework of moral failure.

#### De-exceptionalizing anxiety through shared suffering

Elsewhere in these diaries, anxiety is reframed not as an isolated personal affliction but as one instance of the pressures, disappointments, and losses understood to characterize ordinary human life. In this narrative logic, what is reduced is not suffering itself, but its sense of singularity. By placing anxiety alongside more widely shared forms of strain, narrators weaken the impression that it marks a uniquely personal misfortune. Anxiety thus appears less as a private anomaly than as one expression of a broader condition of social and human vulnerability.

In self-healing diaries, individuals with anxiety disorder often reflect on their condition by referencing the lives of others. Extending the logic of limited individual agency, narrators reframe illness as a universal human condition, establishing a positioning of “I am not special.” The Buddhist phrase “all living beings suffer” frequently appears in such narratives, emphasizing suffering as structural rather than individual.

Patients often adopt a third-person perspective to observe the structural pressures embedded in Chinese society, thereby constructing a social root of anxiety. Discursive images such as Confucian discipline and economic anxiety appear repeatedly. For example, C3 cited a psychiatrist’s comment about rising outpatient visits: “The doctor told me, everyone has some level of anxiety.” B11 expressed recognition of the widespread mental burden associated with adult life: “Who in this society doesn’t have mental health issues? Adults who are overly optimistic are the abnormal ones.” In these accounts, anxiety is not erased, but repositioned within a social and human landscape in which suffering is understood as widely shared rather than uniquely one’s own.

#### Relativizing anxiety against the horizon of death

At its furthest point, this de-exceptionalizing logic places anxiety against the horizon of death, where its threat is no longer narrated as absolute. In this framing, death functions as a limit point that recalibrates the scale of suffering: anxiety is not denied, but it is no longer presented as the worst or final thing that can happen. What is reduced here is not distress itself, but the absolute force of threat attached to it. By placing anxiety within a broader horizon of finitude, these narratives weaken its catastrophic singularity and render it more reconcilable within life.

Many narrators adopt a survivor identity. B17 expressed gratitude for being alive: “Don’t complain about getting this illness—some people die just tripping on the street.” B7 recalled multiple near-death-like experiences and came to embrace a new life philosophy: “Luckily, it wasn’t real death. As long as I’m alive, there’s unlimited possibility. There’s always a way forward.” In these accounts, survival becomes a resource for relativizing anxiety. Framed in contrast with death, illness is no longer narrated primarily as catastrophe, but as something that can be endured, learned from, and lived through.

Some patients take the idea further by using death as a philosophical reference. D1 called death: “Our best teacher.” Through repeated clinic visits, he witnessed breakdowns and suicidal patients. He wrote: “It’s death that taught me what really matters—it helped me find myself. That’s why I’m so calm now.” Here, proximity to death generates a reordered value system in which anxiety is no longer the ultimate enemy to be defeated, but one experience among others through which life acquires clarity, seriousness, and meaning.

### Opportunity narratives: recasting anxiety as a meaningful challenge

Opportunity narratives normalize anxiety by recasting it as a meaningful challenge and a possible pathway to growth. Rather than treating anxiety as a purely negative interruption, self-healing diaries in this mode frame it as a trial through which strength, agency, and renewed orientation may be discovered. What is emphasized here is not the disappearance of suffering, but the possibility that suffering need not be meaningless. By likening anxiety to other demanding yet familiar life challenges and by presenting treatment as a site of effort and transformation, these narratives integrate it into a forward-looking account of self-development. In this sense, normalization takes the form of revaluation: anxiety is repositioned not simply as something to be endured, but as an experience that may be worked through, used, and made consequential within an ongoing life project.

#### Objectifying anxiety as depletion

Opportunity narratives often begin by objectifying anxiety as a form of depletion. Unlike purely affective venting, these accounts describe suffering in a calm and analytic register that renders anxiety assessable in terms of cost, interruption, and wasted effort. What is emphasized here is not only that anxiety hurts, but that it drains time, energy, and possibility in ways that justify action and change. By construing anxiety as an inefficient and externalized burden, these narratives establish the first step in a growth-oriented logic: before anxiety can become a challenge to be worked through, it must first be named as something that cannot simply be merged with or passively endured.

D4 recalled: “I didn’t know why I was sick or what the medication did—doing nothing just drained me.” B14 expressed similar distancing: “I feel awful, but should I just wait for it to pass? I can’t merge with it.”

Here, anxiety is reframed as wasted energy or ineffective struggle. A9 noted that fear of insomnia wasn’t the issue—“anxiety itself is the culprit.” D4 called his half-year leave from school a “waste of life”, stating: “Once I felt it was wasting my time, I wanted to kick it away.” This cost–benefit framing redefines anxiety as an inefficient external disruption. By casting anxiety as something that consumes life without producing value, these narratives justify the need to confront it rather than merely endure it.

#### Reframing the self as capable

Building on this framing of anxiety as a costly and externalized burden, narrators begin to reframe themselves as capable of confronting it rather than being defined by it. Opportunity narratives in this mode do not present strength as something newly bestowed by illness; instead, they retrospectively identify resources that were already present but had not yet been fully recognized. What is emphasized here is not the elimination of vulnerability, but the recovery of agency within it. By narrating the self as already capable of endurance, judgment, and action, these accounts transform anxiety from a sign of inadequacy into an occasion for asserting competence.

Narratives of anxiety treatment often involve a reconstruction of self-identity (Thoits, 2013). B10 stated: “This illness is about cognition—no one can help you.” Patients emphasize that the ability to solve problems independently was not newly gained through therapy, but had long existed, waiting to be activated. For instance, C15, raised by her grandmother after her parents’ divorce, wrote: “My dad visited once a year, but it was fine. I could live well on my own. I’d cry it out and move on.”

By narrating illness as a proof of capability, patients construct a self-image aligned with dominant ideals of success and resilience. A8 rejected the assumption that anxiety reflects poor stress tolerance: “Some people don’t understand. They say I have a weak mindset, like I’ve never faced adversity.” In these accounts, anxiety no longer signifies weakness alone; it becomes a site through which competence, endurance, and self-direction are retrospectively affirmed.

#### Assigning illness a positive value

Once the self is reframed as capable of confronting anxiety, some narrators go on to present illness itself as carrying a positive value. Opportunity narratives in this mode are organized by the conviction that suffering should not remain meaningless: anxiety is reframed not merely as something to survive or confront, but as something that can mark a turning point, confer insight, or justify growth. What is emphasized here is not suffering as desirable, but suffering as consequential. By attributing value to illness, these narratives complete a shift from depletion and challenge to significance, allowing anxiety to be integrated into a positive account of self-development.

As Sontag (1978) noted, illness is often culturally framed as misfortune. Yet some patients construct counter-discourses. C12 wrote: “Only the lucky get this illness. It’s a gift from above. Ugly on the outside—but rich inside.” Rather than deepening pessimism, the low prevalence of anxiety is reframed as a rare and valuable trial, offering symbolic capital.

Building on this, treatment is described as a fast-track to growth. D2 used a gaming metaphor: “Fighting small monsters gives basic rewards. A big monster? That’s your chance to level up.” C5 added: “We’re still young—there’s room to make mistakes.” In this framing, anxiety fits within a logic of controlled risk, resonating with youth culture’s “challenge-reward” mode. Rather than denying pain, such narratives assign it a future-oriented value: illness becomes meaningful insofar as it is made to count for something beyond suffering itself.

## Discussion

This study shows that anxiety is narratively normalized on Xiaohongshu not by being denied or trivialized, but by being repositioned within ordinary frameworks of self-understanding and daily life. What becomes “ordinary” in these self-healing diaries is not anxiety as such, but the place anxiety is given in relation to the body, everyday routines, shared vulnerability, and future-oriented self-development. The recurrent narrative modes identified here therefore suggest that normalization is not a single cognitive move, but a set of patterned narrative operations through which anxiety is made more acceptable, livable, de-exceptionalized, or meaningful. In this respect, patient-authored digital diaries do not merely express distress; they organize anxiety into communicable and socially intelligible forms of experience (Frank, 1995; Sharf and Vanderford, 2003). Their public circulation on social media further underscores narrative as a practical mode of meaning-making rather than a purely stylistic form (Gray, 2009).

The first three modes—acceptance, coexistence, and compromise—normalize anxiety through distinct but related routes: acceptance reduces its catastrophic force, coexistence integrates it into the routines and temporal continuity of everyday life, and compromise de-exceptionalizes it by relocating it beyond personal culpability and within broader conditions of shared vulnerability. Taken together, these modes suggest that normalization is less a matter of “thinking positively” than of re-situating distress within intelligible frames that weaken its singularity and threat. In this sense, the self-healing diaries analyzed here resemble communicative processes through which a “new normal” is constructed after disruption to health and selfhood (Genuis and Bronstein, 2017). They also support scholarship showing that illness narratives may participate in the reconstruction of selfhood and identity after disruption (Cooper, 2021; Gunning and Koenig Kellas, 2024).

Opportunity narratives follow a different route. Rather than primarily reducing the salience of anxiety or absorbing it into existing routines, they revalue suffering by narrating anxiety as a meaningful challenge and a possible site of transformation. In this respect, they come closest to what Frank (1995) describes as quest-like illness narratives, in which suffering is not simply endured but interpreted as yielding insight, orientation, or growth. Yet this revaluation does not amount to a celebration of suffering as such. Instead, they refuse the possibility that suffering should remain meaningless. Anxiety becomes “ordinary” here not because it is made small, but because it is reinserted into a forward-looking life project where challenge, self-work, and consequence remain possible. Such revaluation, however, should not be romanticized. As Wilkinson (2013) cautions, cultural demands to transform suffering into value may themselves become normative burdens.

The predominance of affirmative and integrative narratives in this corpus should also be understood in relation to platform conditions. Xiaohongshu is a lifestyle-oriented, public, and highly visible platform structured by relatability and self-presentation. The self-healing diaries examined here are therefore not only accounts of lived experience; they are also audience-oriented acts of public self-disclosure through which users present themselves as coping, reflective, and moving forward. They remain accounts of lived experience, but should also be understood as a particular narrative formation shaped by public visibility, platform norms, and the selection effects of the “self-healing diary” keyword and the “most popular” ranking. In this sense, the relative absence of more despair-oriented or crisis-centered narratives in the sample should not be read as evidence that such forms are unimportant online. Rather, it indicates that Xiaohongshu rewards certain narratable and shareable forms of anxiety talk more than others. This point is consistent with research showing that some forms of mental distress become difficult to narrate in ordinary communicative settings and may therefore appear differently across platforms (Yeo, 2021). It also resonates with recent work on Xiaohongshu showing that mental-health-related posts are shaped by platformed self-presentation and identity construction (Gu and Gao, 2025).

These findings refine how mental-health communication may be understood in digital environments. The significance of these diaries lies less in any direct clinical function than in making suffering intelligible in public, peer-oriented, and digitally mediated settings. As patient-authored accounts become visible, shareable, and discussable, they can function as forms of experiential knowledge that complicate epistemic asymmetries between clinical expertise and lived experience (Rosen, 2021). This perspective is compatible with person-centered approaches to mental-health care, which require attention not only to symptoms and treatment adherence but also to how patients interpret distress, value care, and narrate recovery or non-recovery (Dixon et al., 2016). In Chinese digital contexts, such peer-oriented narrative exchange may also resemble forms of mutual advice-giving and illness reinterpretation observed in other online mental-health communities (Pun and Yu, 2023; Li and Xu, 2023; Li and Xu, 2025a).

Several limitations should be noted. First, the corpus was drawn from a visible and publicly shareable subset of Xiaohongshu posts and therefore cannot represent all online anxiety discourse in China. Second, the study focuses on authored posts rather than interactional exchanges in comment threads, and thus cannot capture how meanings are negotiated dialogically after publication. Third, because the analysis is restricted to one platform and one narrative label—“self-healing diary”—the findings should be understood as specific to a platformed genre of public self-disclosure. Future research could compare diary-like posts with anonymous forums, crisis narratives, or other platform environments in order to examine how different communicative settings shape what forms of anxiety become sayable, ordinary, or meaningful. Such comparisons would also extend recent work on Chinese online depression communities, where identity reconstruction and collective meaning-making have been shown to vary across digital settings (Li and Xu, 2025b; Xu and Li, 2023).

## Data Availability

The data analyzed in this study is subject to the following licenses/restrictions: the dataset is not publicly available because it consists of sensitive mental-health self-disclosures drawn from publicly accessible social media posts. Although the posts are publicly accessible, they may remain identifiable in context. To protect participants’ privacy, the underlying data are therefore not shared publicly. Requests to access these datasets should be directed to Xudong Zhou, affirmation11@163.com. Requests may be directed to the corresponding author and will be considered on a case-by-case basis, subject to ethical and privacy restrictions.
